# HMGA1 down-regulation is crucial for chromatin composition and a gene expression profile permitting myogenic differentiation

**DOI:** 10.1186/1471-2121-11-64

**Published:** 2010-08-11

**Authors:** Jan Brocher, Benjamin Vogel, Robert Hock

**Affiliations:** 1 Department of Biological Sciences, National University of Singapore, 14 Science Drive 4, Block S1A, Level 6, 117543, Singapore; 2 Biocenter, University of Wuerzburg, Am Hubland, D-97074 Wuerzburg, Germany

## Abstract

**Background:**

High mobility group A (HMGA) proteins regulate gene transcription through architectural modulation of chromatin and the formation of multi-protein complexes on promoter/enhancer regions. Differential expression of HMGA variants has been found to be important for distinct differentiation processes and deregulated expression was linked to several disorders. Here we used mouse C2C12 myoblasts and C2C12 cells stably over-expressing HMGA1a-eGFP to study the impact of deregulated HMGA1 expression levels on cellular differentiation.

**Results:**

We found that induction of the myogenic or osteogenic program of C2C12 cells caused an immediate down-regulation of HMGA1. In contrast to wild type C2C12 cells, an engineered cell line with stable over-expression of HMGA1a-eGFP failed to differentiate into myotubes. Immunolocalization studies demonstrated that sustained HMGA1a-eGFP expression prevented myotube formation and chromatin reorganization that normally accompanies differentiation. Western Blot analyses showed that elevated HMGA1a-eGFP levels affected chromatin composition through either down-regulation of histone H1 or premature expression of MeCP2. RT-PCR analyses further revealed that sustained HMGA1a expression also affected myogenic gene expression and caused either down-regulation of genes such as *MyoD, myogenin, Igf1*, *Igf2*, *Igfbp1-3 *or up-regulation of the transcriptional repressor *Msx1*. Interestingly, siRNA experiments demonstrated that knock-down of HMGA1a was required and sufficient to reactivate the myogenic program in induced HMGA1a over-expressing cells.

**Conclusions:**

Our data demonstrate that HMGA1 down-regulation after induction is required to initiate the myogenic program in C2C12 cells. Sustained HMGA1a expression after induction prevents expression of key myogenic factors. This may be due to specific gene regulation and/or global effects on chromatin. Our data further corroborate that altered HMGA1 levels influence the expression of other chromatin proteins. Thus, HMGA1 is able to establish a specific chromatin composition. This work contributes to the understanding of how differential HMGA1 expression is involved in chromatin organization during cellular differentiation processes and it may help to comprehend effects of HMGA1 over-expression occurring in malign or benign tumours.

## Background

Chromatin provides a platform to regulate gene expression during several biological processes such as cellular differentiation events. Epigenetic programs involve DNA methylation patterns and/or stable modifications of histone tails [1,2]. Most if not all chromatin proteins associating with nucleosomal chromatin bind only transiently and are part of dynamic networks that regulate chromatin organization and function. High mobility group (HMG) proteins are members of these dynamic networks [3]. All members of the three HMG-families are considered as architectural chromatin proteins. Nevertheless, each family or even each family member play distinct roles in chromatin function [3,4].

The mammalian HMGA family consists of four members. Alternative splicing of the HMGA1 transcript gives rise to three variants, HMGA1a, 1b, and 1c while HMGA2 is encoded by a separate gene. Proteins of the HMGA family are characterized by conserved DNA-binding domains, the AT-hooks, and an acidic C-terminal tail [3]. HMGA proteins bind to AT-rich DNA which is considered to be the major reason for their concentration in heterochromatin [3,5,6]. HMGA proteins affect the expression of many genes through architectural remodeling of the chromatin structure and by stabilizing nucleoprotein complexes called enhanceosomes built on promoter/enhancer regions [7,8]. In addition, HMGA proteins are part of further chromatin complexes, as has been shown for the pre-replication complex [9] and are able to influence the structure and function of large chromatin domains [8,10].

During development HMGA proteins are highly expressed in early embryos and undifferentiated cells but are absent in differentiated cells [4]. Thus, a regulated HMGA expression is important for proper cell function and differentiation. High expression levels are found in many tumors and correlate with tumor malignancy [11], are linked to deregulated oncogenes and contribute to genomic instability by inhibition of proper nucleotide excision repair [12]. Several reports indicated that HMGA proteins influence expression of genes in a cell type specific manner [4].

Loss of *Hmga1 *or *Hmga2 *gene function affects specific differentiation processes [4]. *Hmga1 *knockout mice develop type 2 diabetes due to a reduced expression of the insulin receptor [13], cardiac hypertrophy and myelo-lymphoproliferative disorders [14]. HMGA2 was shown to be crucial for cardiogenesis through regulating the gene *Nkx2.5*, a cardiogenic key transcription factor [15]. A pygmy phenotype of mice is caused by a disrupted *Hmga2 *gene and characterized by drastic reduction of fat tissue and a deficient spermatogenesis [16,17].

Here, we demonstrate that after induction of myogenesis in C2C12 cells down-regulation of HMGA1 proteins is an early and required step allowing the progression of the myogenic program. Sustained HMGA1a expression prevented myogenic differentiation and altered the chromatin composition through interfering with the expression of myogenic genes and other architectural chromatin proteins.

## Results

### Down-regulation of HMGA1 proteins during cellular differentiation

**Figure 1:**
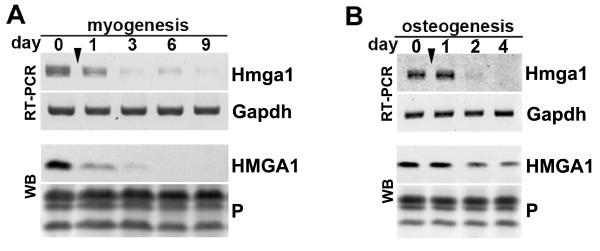
**Differential expression of HMGA1 proteins during C2C12 cell differentiation**. **(A) **Down-regulation of HMGA1 during myogenic differentiation of C2C12 cells as analyzed by PCR and Western blotting (WB). For Western blots proteins of 1.5 × 10^5 ^nuclei were separated on a 15% SDS-polyacrylamide gel. For RT-PCR 1 μg of total RNA was used to produce cDNAs for the PCR reaction. For PCR identical amounts of cDNA were used. Time points of analyses are indicated in days. The arrowhead marks the time point of induction. Day 0 denotes non-induced myoblasts. *Gapdh *expression served as a control for the reverse transcription in RT-PCR experiments. As a control for loading and Western blotting Ponceau staining (P) of core histones is shown. **(B) **Down-regulation of HMGA1 expression during osteogenesis. Osteogenesis in C2C12 cells was induced with 0.5 μg/ml BMP2 (arrowhead) and HMGA1 expression was analyzed at day 0 and on days 1, 3 and 4 after by RT-PCR (RT) and Western blot (WB) as described in (A).

Murine C2C12 cells are committed cells that initiate muscle differentiation upon growth factor withdrawal or initiate osteogenesis upon addition of the growth factor BMP2. After induction of the myogenic program major morphological changes in C2C12 cells occurred on day 1-3 (cellular elongation) and on days 6-9 (cell fusion and myotube formation). Analyses of *Hmga1 *expression by RT-PCR and Western blots revealed an immediate down-regulation of *Hmga1 *expression after induction of myogenic differentiation reaching low or undetectable levels on day 3 and subsequent time points during differentiation, respectively (Fig. 1A). Similarly, induction of osteogenesis by BMP2 also caused down-regulation of *Hmga1 *mRNA with a delayed onset compared to the down-regulation during myogenesis. Interestingly, HMGA1 protein levels remained well detectable even after 4 days of osteogenic differentiation (Fig. 1B). The persistence of HMGA1 protein compared to the absence of detectable mRNA might result from different protein stabilities dependent on the cellular context during the two differentiation pathways. These data support that *Hmga1 *expression is only prominent in undifferentiated cells but down-regulated after the initiation of differentiation upon external stimuli.

### Characterization of C2C12 cells stably expressing HMGA1a-eGFP

**Figure 2:**
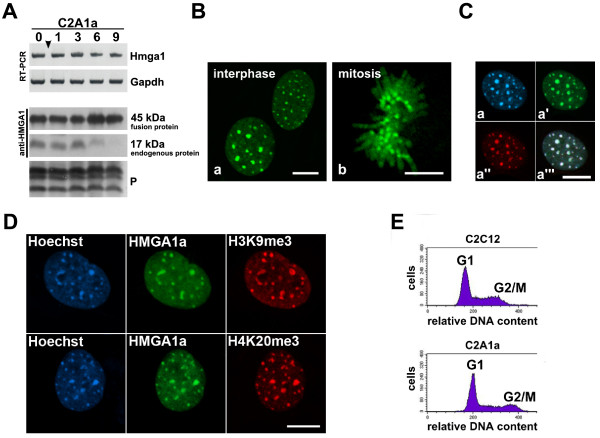
**Characterization of the HMGA1a-eGFP over-expressing cell line C2A1a**. **(A) **Sustained expression of HMGA1a-eGFP in C2A1a cells throughout myogenic induction analyzed by RT-PCR and Western blotting (WB) as indicated. Both, fusion protein (~45 kDa) and endogenous protein (~17 kDa) were detected using an HMGA1-specific antibody. Note the sustained expression throughout myogenic induction. Loading controls are as mentioned in Fig. 1. (B) Localization of HMGA1a-eGFP in living C2A1a cells. Note the concentrated localization in heterochromatin foci (chromocenters) during interphase (a) and on pericentromeric regions during mitosis (b). Scale bars represent 10 μm. **(C) **Immunolocalization on fixed C2A1a cells using HP1α-specific antibodies. Note the colocalization of HMGA1a-eGFP (a') and HP1α (a'') in the chromocenters of C2A1a cells. DNA was stained with Hoechst (a). An overlay of a-a'' is shown in a'''. The bar represents 10 μm. **(D) **HMGA1a-eGFP (green) colocalizes with the H3K9me3 and H4K20me3 specific immunolocalizations (red) in C2A1a cells. DNA was stained with Hoechst. The bar represents 10 μm. **(E) **Cell cycle phases are unaffected in the C2A1a cell line. DNA from C2C12 and C2A1a was stained with propidium iodide and 20,000 cells from each cell line were analyzed by FACS. Cell numbers (counts) are plotted against the relative DNA content of the cells. Phase distribution was analyzed with modfit Lt3.1. 56.14% of C2C12 cells were in G1 phase, 29.54% in S phase, and 14.33% in G2 phase. In C2A1a cells 60.95% of the cells were in G1 phase, 24.43% in S phase, and 14.63% in G2 phase.

To assess whether *Hmga1 *down-regulation is required for cell differentiation we generated C2C12 cells stably over-expressing HMGA1a-eGFP (C2A1a cells). As previously shown, HMGA1a-eGFP fusion proteins behave like endogenous proteins [6]. HMGA1a-eGFP expression was constant throughout the entire time the C2A1a cells were cultured under myogenic induction conditions (Fig. 2A). Western blots revealed that the over-expression of exogenous HMGA1a-eGFP in C2A1a cells resulted in a prolonged expression of endogenous HMGA1. The latter was still detectable six days after culturing C2A1a cells in differentiation medium while HMGA1 was undetectable in C2C12 wild type cells already 3 days after induction. Endogenous HMGA1 and exogenous HMGA1-eGFP were detected in parallel by an HMGA1-specific antibody to compare relative expression levels (Fig. 2A). Semi-quantitative densitometric evaluation of Western blots using ImageJ indicated a ~2.6-fold over-expression of HMGA1 proteins as compared to endogenous HMGA1 in wild type myoblasts.

In living C2A1a cells, HMGA1a-eGFP preferentially localized throughout the cell cycle in heterochromatin foci which represent pericentromeric regions fused into larger entities called chromocenters (Fig. 2B). In interphase cells it colocalized with markers for heterochromatin such as HP1α, histone H3 trimethylated at K9 or histone H4 trimethylated at K20 (Fig. 2C, D). In agreement with previous data that linked increased HMGA levels to enhanced cell proliferation, we counted a 2.6-fold increase in the C2A1a cell number 24 hours after seeding the same amount of C2C12 and C2A1a cells. FACS analyses revealed a similar cell cycle stage distribution of the transformed and parental cells (Fig. 2E).

### Stable expression of HMGA1a prevents myogenic differentiation of C2C12 cells

**Figure 3:**
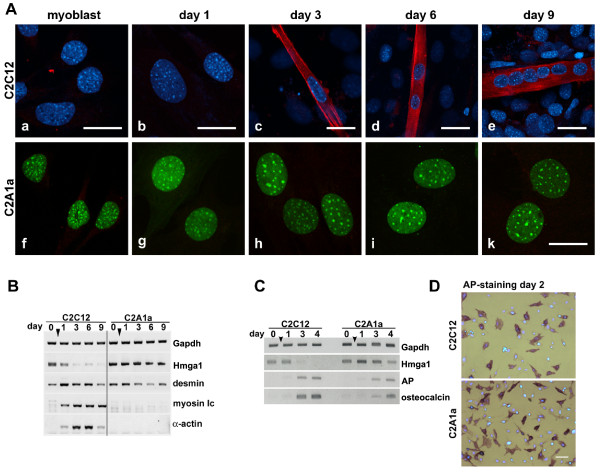
**HMGA1a over-expression prevents myogenic differentiation**. **(A) **Immunofluorescence localization of myosin (red) in C2C12 (a-e) and in C2A1a cells (f-k) before (myoblast) and after induction (days 1 to 9). All bars represent 20 μm. Myotube formation was only observed in wild type C2C12 cells. In pictures a-e DNA staining by Hoechst is shown in blue, respectively. HMGA1a-eGFP is shown in green. **(B) **RT-PCR analysis to compare the expression of the myosin light chain (*myosin lc*) and *α-actin *in C2C12 cells (left) and in C2A1a cells (right) as described in Fig. 1. Expression of *Hmga1, Gapdh *and *desmin *are shown as controls. **(C) **RT-PCR to analyze expression profiles of marker genes for osteogenesis. Genes analyzed were *alkaline phosphatase *(AP) and *osteocalcin*. *Gapdh *expression is shown as control. HMGA1a over-expression did not affect *AP *and *osteocalcin *transcription. **(D)** Alkaline phosphatase activity as marker for osteogenesis of C2C12 and C2A1a cells on day 2 of differentiation (bright field images). AP activity was visualized using NBT/BCIP staining. Shown are overlays of bright field images and fluorescence images with corresponding DNA staining. Scale bar is 50 μm.

To compare myogenesis in C2C12 and C2A1a cells we used immunolocalization experiments as well as RT-PCR. Immunofluorescence indicated that C2A1a cells, but not C2C12 cells, failed to fuse and to form myosin positive myotubes (Fig. 3A). We further tested the expression of α-actin and myosin light chain mRNA as a marker for myogenic differentiation. In C2C12 cells, transcripts of both markers were detectable by RT-PCR shortly after induction of differentiation (Fig. 3B, left). In contrast, they were absent in C2A1a cells grown for at least 9 days in differentiation medium (Fig. 3B, right). On the contrary, as monitored by expression of *alkaline phosphatase *and *osteocalcin*, early osteogenesis was not affected (Fig. 3C, D). Together these data demonstrate that sustained expression of HMGA1a does not interfere with early osteogenic events but specifically impairs myogenesis in C2C12 cells.

### Sustained HMGA1a expression prevents chromocenter remodeling

**Figure 4:**
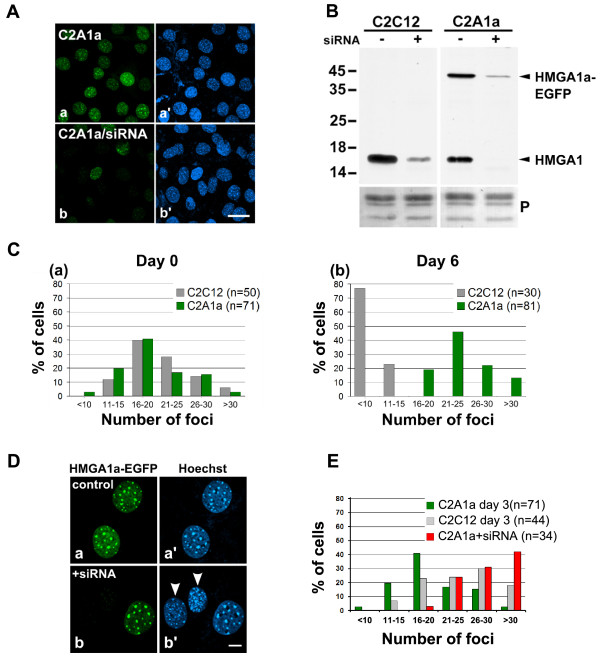
**HMGA1a over-expression interferes with chromatin organization during myogenesis**. **(A) **Depletion of HMGA1a-eGFP after HMGA1a-siRNA treatment is visual through the loss of eGFP fluorescence in C2A1a cells (b). Mock transfected C2A1a cells are shown in a. The corresponding Hoechst stained images are shown in a' and b'. The bar represents 20 μm. **(B) **Western blot analysis showed considerable depletion of endogenous and eGFP-tagged HMGA1a proteins, respectively, 12-24 hours after siRNA treatment of C2C12 and C2A1a cells. Ponceau staining (P) of the core histones is presented as loading control. Protein molecular mass is indicated in kDa. **(C) **Heterochromatin foci (chromocenters) in C2C12 and C2A1a cells were quantified (n = cell number) and plotted as indicated. (a) In myoblasts (day 0) the number of chromocenters is identical in both cell lines. (b) During terminal muscle differentiation of the C2C12 cells, the number of heterochromatin foci decreases due to chromocenter clustering (grey columns) whereas the chromocenter number in C2A1a cells remains comparable to the number in myoblasts (green columns). **(D) **C2A1a control cells (a) and C2A1a cells 12-24 hours after siRNA treatment (b). HMGA1 depletion, indicated by absence of HMGA1a-eGFP, results in a higher number and a reduced size of chromocenters (arrowheads) (b'). The bar represents 10 μm. **(E) **Depletion of HMGA1 by siRNA in C2A1a myoblasts increased the chromocenter number (red columns) compared to C2A1a control cells (green columns). A similar chromocenter dissociation was detected in C2C12 wild type cells on differentiation day 3 (grey columns) prior to fusion during terminal differentiation.

Reorganization of chromatin accompanies cellular differentiation. In C2C12 cells, differentiation associated chromatin reorganization is visual as clustering of chromocenters during terminal differentiation leading to a reduced chromocenter number in differentiated cells [18]. To examine whether variations in HMGA1 levels participate in chromocenter remodeling we compared their numbers in C2C12 cells, C2A1a cells (increased HMGA1 level) and C2A1a cells after HMGA1 knock-down through siRNA (reduced HMGA1 level). Successful knock-down of endogenous HMGA1 and HMGA1a-eGFP was verified by loss of eGFP-fluorescence (Fig. 4A) and by Western blot analyses (Fig. 4B). Number and distribution of chromocenters were found to be almost identical in non-induced C2C12- and C2A1a-myoblasts (Fig. 4C, a). Consistent with Brero et al., [18], reduced chromocenter numbers indicated chromocenter clustering in terminally differentiated C2C12 cells (Fig. 4C, b grey bars). In contrast, even after growing C2A1a cells for 6 days in differentiation medium, the number of chromocenters remained comparable to the number of chromocenters in non-induced cells or even shifted to an increased percentage of cells with increased chromocenter number (Fig. 4C, b). Thus, HMGA1 over-expression prevented chromocenter clustering which occurs normally through terminal differentiation and stabilized a chromocenter distribution comparable to non-induced myoblasts.

We further asked, what happens to the chromocenter organization after HMGA1a knock-down. Therefore, we evaluated the chromocenter number in C2A1a myoblasts that lost their eGFP fluorescence as a marker for HMGA1 knock-down after Hoechst staining (Fig. 4D). Of note, the fraction of cell nuclei with more than 30 chromocenters significantly increased from 2.8% to 42% in cells without eGFP fluorescence (p < 0.001). This suggests that reduced HMGA1 protein level in non-induced C2C12 cells lead to a reduced chromocenter stability. It should be noted that chromocenter dissociation was observed transiently between 12-24 hours after HMGA1 knock-down through siRNA treatment (Fig. 4E). Comparable chromocenter dissociation was observed in C2C12 cells around day 3 of differentiation when endogenous HMGA1 is down-regulated (Fig 4E, grey bars) indicating that transient chromocenter dissociation naturally and transiently occurs prior to chromocenter clustering. Together this suggests that HMGA1a over-expression stabilizes chromocenters and prevents their remodeling prior to clustering during terminal differentiation.

### HMGA1 over-expression alters global chromatin composition

**Figure 5:**
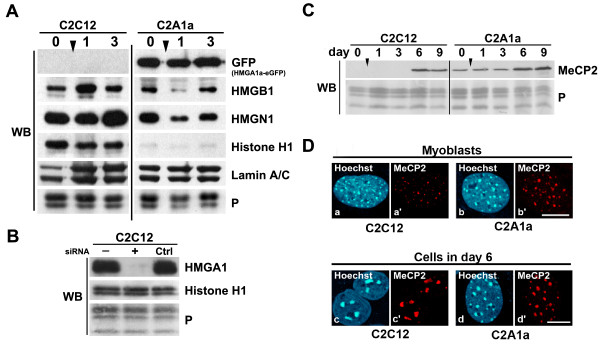
**HMGA1a over-expression alters the chromatin composition**. **(A) **Western blot analyses comparing the expression of HMGB1, HMGN1, and histone H1 in C2C12 cells and C2A1a cells in myoblasts (day 0) and on days 1 and 3 of differentiation. The antibody used [43] recognizes all histone H1 variants. Proteins of 1.5 × 10^5 ^cells were analyzed in 15% SDS-PAGE. Lamin A/C expression and Ponceau S staining are shown as loading controls. **(B) **Histone H1 levels are unaffected in C2C12 cells after HMGA1 knock-down. Western blot was as described in Fig. 5A. Antibodies used are indicated. Histone H1 antibody used was directed against all H1-sub-variants (Abcam). Cells were untreated (-), siRNA treated (+) or transfected using siRNA control oligos (Ctrl). Ponceau S staining is shown as loading control. **(C) **Western blot analysis comparing MeCP2 expression in C2C12 and C2A1a cells. Note the premature MeCP2 expression in C2A1a cells. **(D) **Immunofluorescence localization of MeCP2 in C2C12 and C2A1a cells. MeCP2 is hardly detectable in C2C12 myoblasts (a') but accumulates in chromocenters of C2A1a cells (b). MeCP2 is concentrated in fused chromocenters in C2C12 cells (c') and is accumulated in chromocenters of C2A1a cells on day 6 after induction (d'). Note the absence of chromocenter clustering. Corresponding DNA staining is shown in a, b, c and d, respectively. Scale bars represent 10 μm.

HMG proteins have been shown to globally affect chromatin organization and function as players in dynamic networks through regulating the access of other factors and modulators to chromatin [3]. Little is known about how HMG proteins affect chromatin composition through affecting expression of other architectural chromatin proteins. We therefore examined by Western blotting how over-expression of HMGA1a influences the expression of HMGB1, HMGN1 and histone H1 during cellular differentiation (Fig. 5A). The expression levels of HMGB1 and HMGN1 were different in C2C12 and C2A1a cells, displaying a slight down-regulation especially at day 1 after induction of C2A1a cells. Notably, histone H1 levels were constantly decreased in C2A1a cells before and throughout induction of myogenesis. In contrast, histone H1 levels remained unaffected after HMGA1a knock-down in uninduced C2C12 cells (Fig. 5B). This suggests that the effect on histone H1 expression only occurs when HMGA1a is over-expressed in C2A1a cells and that the down-regulation of histone H1 may be an indirect effect.

During differentiation of C2C12 cells the heterochromatin associated methyl-CpG-binding protein MeCP2 is highly expressed only during terminal differentiation and involved in chromocenter clustering [18]. In contrast to HMGA1, over-expression of MeCP2 is sufficient to cause chromocenter clustering even in the absence of differentiation [18]. Therefore, we examined MeCP2 expression in more detail. Consistent with Brero et al. [18] we found that MeCP2 expression in C2C12 cells started at day 6 of differentiation (Fig. 5C, left) and only a minor fraction of MeCP2 was localized in chromocenters of myoblasts (Fig. 5D, a'). On day 6 of differentiation MeCP2 was concentrated in fused chromocenters in C2C12 cells (Fig.5D, c').

In contrast, we detected a premature expression of MeCP2 in C2A1a cells (Fig. 5C, right) and MeCP2 was already accumulated in chromocenters of C2A1a myoblasts (Fig. 5D, b'). Nevertheless, as mentioned earlier, chromocenter clustering was prevented in C2A1a cells (Fig. 5D, d'). Thus, HMGA1a over-expression elevates the expression of MeCP2 but also counteracts its capability to cause heterochromatin fusion.

Together, these data demonstrate that changes in HMGA1a levels cause an alteration of the expression of architectural chromatin proteins and are therefore able to modulate global chromatin composition on the level of gene expression.

### HMGA1a over-expression deregulates myogenic gene expression

**Figure 6:**
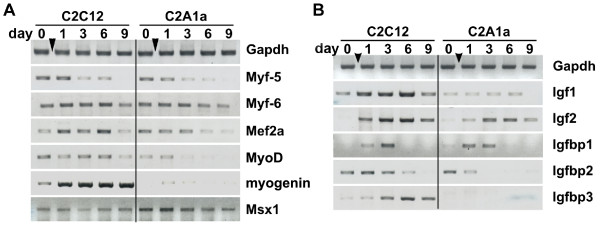
**Sustained HMGA1a-eGFP expression interferes with the expression of myogenic genes**. **(A) **Comparison of expression profiles of myogenic transcription factors in myoblasts (day 0) and during differentiation (days 1, 3, 6, 9 after induction). Gene expression was analyzed by RT-PCR as described in Fig. 1. Genes analyzed were: myogenic factors 5 and 6 (*Myf5 *and *Myf6*), the myocyte enhancer factor 2A (*Mef2a*), myogenic determination gene 1 (*MyoD*), myogenin and homeobox, msh-like 1 (*Msx1*). Note the specific down-regulation of *MyoD *and *myogenin *and the up-regulation of the myogenic inhibitor *Msx1 *in C2A1a cells. As loading control *Gapdh *expression is shown. **(B)** Expression profiles of components of the Igf-system in C2C12 and C2A1a cells at the time points described in (A). Genes analyzed were: insulin-like growth factors 1 and 2 (*Igf1 *and *Igf2*) and Igf binding proteins 1, 2 and 3 (*Igfbp1*, *Igfbp2 *and *Igfbp3*). Note the suppression of *Igf1*, *Igf2*, *Igfbp2 *and *Igfbp3*. *Gapdh *expression is presented as a control.

To examine whether the impaired myogenesis of C2A1a cells could be due to altered expression of myogenic factors we analyzed (by RT-PCR) the expression profiles of the transcription factors myogenic factor 5 and 6 (*Myf5 *and *Myf6*), myocyte enhancer factor 2A (*Mef2a*), the myogenic determination gene 1 (*MyoD*), *myogenin *and the myogenic inhibitor homeobox, msh-like 1 (*Msx1*) (Fig. 6A). Compared to C2C12 cells, the expression of *MyoD *and *myogenin *was significantly suppressed in C2A1a cells. Mef2a seemed to be only slightly down-regulated. In contrast, the myogenic inhibitor *Msx1 *was up-regulated. The expression profiles of other factors involved in myogenic differentiation like *Myf5 *and *Myf6 *(Fig. 6A) remained unaffected by sustained HMGA1a expression.

Besides transcription factors, growth factors such as insulin-like growth factor 1 and 2 (*Igf1 *and *Igf2*) are required for proper myogenesis. Igf binding proteins 1, 2, and 3 (*Igfbp1, Igfbp2, Igfbp3*) further fine tune the bioavailability of Igf1 and Igf2. RT-PCR analyses revealed that *Igf1*, *Igf2*, *Igfbp2 *and *Igfbp3 *were down-regulated in C2A1a cells after induction, indicating that HMGA1a that is present after induction is able to suppress the expression of components of the Igf-system (Fig. 6B).

These data illustrate that a sustained high HMGA1a protein level after induction of myogenesis alters the expression of specific genes crucial for myogenesis and prevents to establish a proper myogenic gene expression profile.

### Knock-down of HMGA1 in HMGA1a over-expressing cells is sufficient to re-initiate myogenic differentiation

**Figure 7:**
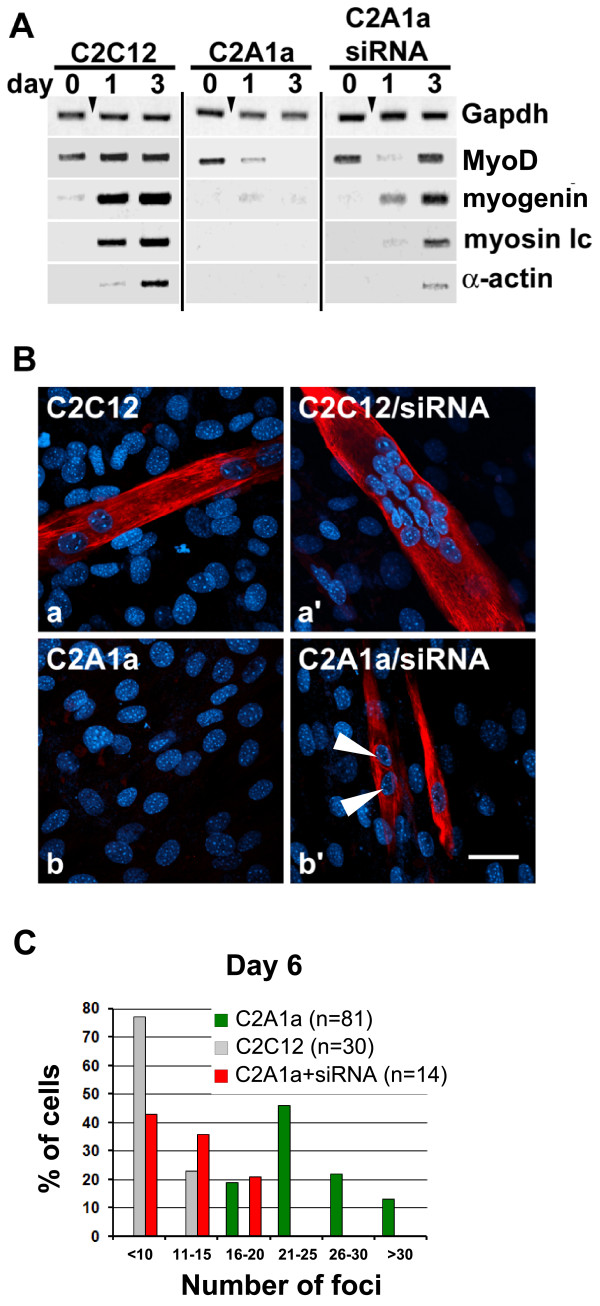
**Recovery of myogenic gene expression in C2A1a cells after siRNA mediated HMGA1 depletion**. **(A) **RT-PCR to analyze myogenic gene expression after HMGA1 knock-down in C2A1a cells. RT-PCR was as described in Fig. 1. Days of analyses are indicated. *Gapdh *expression was used as a control. Note the recovery of *MyoD*, *myogenin*, *myosin lc *and *α-actin *expression on day 3 of differentiation after HMGA1 knock-down in C2A1a cells. **(B) **Immunofluorescence localizations of myosin (red) in C2C12 and C2A1a control cells (a, b) and C2C12 and C2A1a cells treated with HMGA1 siRNA (a', b') on day 6 after induction. DNA was stained with Hoechst (blue). The siRNA treated C2C12 cells (a') differentiate as control treated C2C12 cells (a). Knock-down of HMGA1 in C2A1a cells recovers myotube formation and myosin expression (b'). Note that the number of chromocenters is reduced in these cells indicating chromocenter clustering (arrows). Bar represents 100 μm. **(C) **Chromocenter clustering occurs after HMGA1a knock-down in C2A1a cells. Comparison of chromocenter numbers on day 6 of differentiation in terminal differentiated C2C12 cells (grey columns), C2A1a on day 6 throughout induction of myogenesis (green columns) and myosin positive C2A1a cells after knock-down of HMGA1a on day 6 of differentiation (red columns). The evaluation of chromocenter numbers was as described in Fig. 4C.

We performed siRNA experiments to examine whether HMGA1 knock-down would restore the ability of C2A1a cells to undergo myogenic differentiation. Notably, in C2C12 and C2A1a cells, HMGA1 knock-down through siRNA was not sufficient to initiate the myogenic program and still required induction by serum withdrawal (data not shown). However, siRNA mediated knock-down of HMGA1a in C2A1a cells was sufficient to reactivate the potential of C2A1a cells to enter the myogenic program after induction. RT-PCR revealed regained expression of *MyoD*, *myogenin*, *myosin lc *and *α-actin *on day 3 after induction (Fig. 7A). these data demonstrate that down-regulation of HMGA1a is a crucial pre-requisite for the initiation of the myogenic program after induction and necessary to enable C2C12 cells to establish a specific gene expression profile that is essential for the correct course of myogenic differentiation. Furthermore, knock-down of HMGA1a in C2A1a cells restored myosin expression 3-6 days after induction as well as chromocenter clustering accompanying terminal differentiation (Fig. 7B, arrows and Fig. 7C). This supports that HMGA1a down-regulation is crucial to activate the entire myogenic program including chromatin remodeling during terminal differentiation.

## Discussion

HMGA1 proteins are architectural chromatin proteins known to be preferentially expressed in proliferating embryonic tissues but absent in differentiated cells [4,19]. HMGA1 proteins have been previously implicated in the differentiation of several cell types. For example HMGA1 affects lympho-hematopoietic differentiation of mouse embryonic stem cells [20] and the differentiation of sperm cells [21]. HMGA1 proteins bind to adipocyte-specific promoters and down-regulation has been shown to impair adipocytic differentiation of 3T3-L1 cells [22]. Here we demonstrate that HMGA1 down-regulation is one of the first and essential steps to allow myogenic differentiation of C2C12 cells. In contrast, sustained expression of HMGA1a-eGFP after induction prevents myogenic differentiation. Mechanistically, the inhibition of C2C12 myogenesis is caused by a specific down-regulation of the myogenic key transcription factors MyoD and myogenin and several additional factors that are required to progress myogenesis.

Several mechanisms have been described on how HMGA proteins participate in specific gene expression, for example the formation of enhanceosomes [8], binding to specific promoter regions to remove inhibitory factors and to recruit chromatin remodeling complexes or to interact with other transcription factors (e.g. Smad1/4) [8,15,23,24].

The genes that are specifically targeted by HMGA1a during C2C12 myogenesis remain to be examined. Certainly, the down-regulation of specific myogenic genes through HMGA1a is indirect and may represent downstream effects in myogenic gene activation cascades. For example, the decreased *myogenin *expression is likely caused by the down-regulation of *MyoD *as well as *Mef2a*. The latter was recently shown to be necessary for efficient expression of *myogenin *through the binding to its promoter [25]. *MyoD *in turn might be repressed through up-regulation of its suppressor *Msx1*. Even though elevated *Msx1 *expression in C2A1a cells was just prominent until three days after induction, this initial up-regulation might be sufficient to aggravate the effects of inhibition on the myogenic program. In contrast, it is also conceivable that the differential expression of these genes observed in C2A1a cells is regulated by HMGA1 independently of each other, while affecting the differentiation program in a synergistic manner. Due to this possibility, the promoters of *MyoD*, *myogenin *as well as *Msx1 *are good potential candidates for being direct HMGA1a targets. Other direct candidate genes are those of the Igf-pathway which we found to be suppressed through sustained HMGA1a expression (e.g. *Igf1*, *Igf2*). Several previous reports discussed that Igf-signaling is involved in sugar metabolism [26] and myogenic differentiation [27-29] and Igf1 depletion impairs functional muscle development in mice [30-32]. Supporting that, Igf1 induces *myogenin *expression followed by cell cycle arrest and myogenic differentiation [33]. Depletion of *Igf2 *in C2 cells inhibits *MyoD *expression and abolishes the ability of the cells to express *myogenin *and *myosin *genes [34]. Thus, the observed deregulation of the Igf-signaling through HMGA1a over-expression may cause and/or amplify the lack of key myogenic transcription factors and is in good correlation to our observed inhibition of myogenesis.

Despite specific effects on gene promoters, sustained HMGA1a expression may also affect gene regulation through a more global regulation of chromatin architecture. For example, it has been shown that HMGA1 binds to A/T-rich scaffold attachment regions (SARs) which are thought to organize larger chromatin domains [10]. Previous reports showed that HMGA proteins are preferentially associated with heterochromatin [6,35]. This is supported by the preferential localization of HMGA1a in chromocenters of C2C12 cells.

HMG proteins, histone H1 and many other chromatin proteins are members of a large network of chromatin binding factors that dynamically modulate chromatin architecture through interaction and competition [3]. The function of this network also depends on the availability of HMGA1-interactors and competitors such as histone H1 [3]. HMGA1 proteins were found to induce transcription of previously suppressed plasmid templates by displacement of histone H1 from SAR elements [36]. In support, it was shown that HMG proteins in general compete for chromatin binding with histone H1 in living cells [37]. The significantly decreased levels of histone H1 in HMGA1a over-expressing C2C12 cells demonstrate a shift in the regulatory equilibrium of those two chromatin proteins, favoring HMGA1 binding to previously H1-suppressed sites. This could lead to the modulation of the structure and activity of large chromatin loops and thus affect myogenic gene expression.

The massive down-regulation of histone H1 was surprising. This raises the question how the cells could tolerate this. However, besides histone H1 additional chromatin proteins such as HMGB1, HMGN1 and MeCP2 were also misregulated. This indicates that the entire chromatin composition is altered and that the loss of histone H1 may be compensated by other chromatin proteins like HMGB1 [38] or other differentiation specific histone H1 variants which are not detected by the H1 antibodies used. Within this context it is important that the over-expression of HMGA1a-eGFP prevented chromocenter remodeling and thus global chromatin reorganization normally accompanying differentiation. Interestingly, remodeling of chromocenters was completely recovered after knock-down of HMGA1a in C2A1a cells which was visual through regained chromocenter clustering during the restored terminal differentiation. Notably, the protein MeCP2, which stabilizes chromocenter organization in differentiated cells, was up-regulated in C2A1a cells. MeCP2 dynamically interplays with HP1 proteins, and it was suggested that this interaction in turn stabilizes chromatin organization [39]. Consistently, premature MeCP2 expression in HMGA1a over-expressing C2A1a cells could therefore increase and stabilize the HP1 concentration on chromatin which in turn could stabilize a chromatin structure that prevents expression of genes relevant for myogenic differentiation.

## Conclusions

We have shown that down-regulation of HMGA1 chromatin proteins is crucial to initiate the myogenic program after induction of C2C12 differentiation. Thus, we provide an example how differential expression of HMGA1 proteins is involved in differentiation processes. After induction, sustained HMGA1a expression alters the transcription of genes that are relevant for initiation and the proper course of myogenic differentiation. Both, specific gene regulation and global effects on chromatin may contribute to this deregulated gene expression. Global effects involve deregulated expression of other chromatin proteins such as histone H1 and MeCP2, leading to a modified chromatin composition. More generally, these latter data propose that altered levels of HMGA1 proteins are connected to the expression of architectural chromatin proteins and thus are able to establish a specific chromatin composition.

This report contributes to the understanding of how the differential expression of HMGA1 proteins is involved in chromatin organization in undifferentiated cells and during differentiation processes. Furthermore, it may help to comprehend possible mechanisms of HMGA function in malign and benign tumours that over-express HMGA proteins.

## Methods

### Cell culture and differentiation

C2C12 cells were cultured in growth medium containing DMEM 4.5 g/L glucose (Gibco/Invitrogen), supplemented with 100 units/ml Penicillin, 100 μg/ml Streptomycin, 2 mM L-glutamine and 10% FCS. Myogenic differentiation was induced with differentiation medium (DM) containing DMEM 4.5 g/L glucose, 100 units/ml Penicillin, 100 μg/ml Streptomycin, 2 mM L-glutamine and 2% horse serum (Gibco/Invitrogen). Osteoblast differentiation was initiated by addition of 0.5 μg/ml BMP2. C2C12 cells stably expressing HMGA1a-eGFP constructs were generated by retroviral transduction as shown below. After isolation, cell clones were grown and differentiated as described above.

293T cells were grown in DMEM 1 g/L glucose, supplemented with 100 units/ml Penicillin, 100 μg/ml Streptomycin and 10% FCS.

### Cell transfections and production of ecotropic viruses for transduction of C2C12 cells

To produce C2C12 cells stably expressing HMGA1a-eGFP, cells were infected with ecotropic retroviruses. To achieve this, 293T cells were transfected transiently with pHIT60 [40] including *gag-pol*, pcziMEE bearing the sequence of a mouse specific envelope protein (*env*) and pLTR-HMGA1a-eGFP. The last two plasmids were derived from pLTR-eGFP via deletion of the eGFP sequence and insertion of HMGA1a-eGFP. 293T triple transfection of retroviral vectors was performed with MATra (IBA) using 6 μg of total plasmid DNA (2 μg each plasmid). Medium was changed 24 h after transfection and 10 mM Na-butyrate (Sigma) was added. 8 h after Na-butyrate treatment cells were washed and grown for further 24 h in normal growth medium. Supernatant was sterile filtered (45 μm) and mixed with polyprene (Sigma) to a final concentration of 8 μg/ml. The mixture was added to the C2C12 cells. After 8 h, C2C12 cells were washed once in PBS and grown for 12 h in growth medium. Positive colonies were manually selected using fluorescence microscopy.

### Alkaline phosphatase staining

Cells grown on cover slips were fixed for 15 min in 2% formaldehyde/PBS. After fixation the cells were washed 2 times in ALP buffer (100 mM Tris, pH 9.5; 100 mM NaCl; 50 mM MgCl2) and incubated with 30 μl of ALP buffer containing 4.5 μl NBT (100 mg/ml in 70% dimethylformamide; Roche) and 3.5 μl BCIP (50 mg/ml in dimethylformamide; Roche) until AP staining was visualized (~5-10 min). After two additional washing steps, coverslips were mounted in Mowiol as described [41].

### FACS analysis

C2C12 and C2A1a cells were grown in a 60 mm culture dish to ~80-90% confluence. Cells were removed from the culture dish, washed in PBS for 10 min and pelleted by centrifugation. The washing step was repeated twice with a pre-warmed 2% FCS/PBS solution. Finally, cells were resuspended in 1 ml 2% FCS/PBS. Then 3 ml ice cold ethanol was added dropwise. Cells were fixed for 1 h at 4°C, washed twice in PBS and resuspended in 1 ml PBS. 50 μg of propidium iodide and 200 μg of RNAse (Sigma) were added and incubated for 30 min at 37°C. FACS analyses were carried out using a BD FACScan cell sorter and CellQuest Pro Software.

### siRNA treatment

RNA oligos (Biomers) used for siRNA experiments were designed on basis of the genebank number AF285780: mHMGA1a-Ex5-sense, 5'-aagucaccacagcuccaggga-3'; mHMGA1a-Ex5-antisense 5'-ucccuggagcuguggugacuu-3'; mA1a-Ex5-2-sense, 5'- aaggggcagacccaagaaacu-3'; mA1a-Ex5-2 antisense, 5'-aguuucuugggucugccccuu-3'. For siRNA controls the sequence of non-targeting siRNA was used as described [42]. For annealing 30 μl of sense and appropriate antisense oligo (50 μM each) were mixed and 15 μl of 5x annealing buffer (50 mM Tris/HCl, pH 7.5; 100 mM NaCl) was added. The final concentration of the duplex was 20 μM. Annealing was performed in a PCR cycler at 95°C for 2 min and cooling down to 20°C over 60 min. For siRNA transfection 2.5 μl (50 μM) of each siRNA duplex were mixed and transfected into C2C12 cells using TransFectin (BioRad). Knock-down of HMGA1a proteins was analyzed in nuclei isolated 12-24 hours after siRNA transfection by Western blot or by loss of HMGA1a-eGFP fluorescence.

### Immunofluorescence procedures

C2C12 cell lines grown on coverslips were washed in PBS for 5 min, fixed in 2% formaldehyde/PBS for 15 min, washed again and permeabilized for 15 min in 0.1% Triton X-100/PBS. After additional washing steps, cells were incubated in 100 mM glycin/PBS. All antibody incubations were performed in a humidified chamber. Following antibodies were used for immunofluorescence: anti-MeCP2 (1:1000, abcam), anti-H3K9me3 (1:1000, abcam), anti-H3K20me3 (1:500, abcam), anti-myosin (1:500, Sigma), anti-HP1á (1:100, Chemicon). Primary antibodies were incubated over night at 4°C. After two washing steps in PBS, 25 μl of appropriate secondary antibodies were incubated for 20 min. To stain DNA, 10 μl of Hoechst/PBS (5 μg/ml) were added and incubated for further 10 min. After two final washing steps, coverslips were mounted in Mowiol as described. Confocal analyses were performed with a Leica TCS-SP2/AOBS using a HCX Pl APO 63x 1.4 oil immersion objective, using sequential scans and lasers with the appropriate wavelengths.

### RT-PCR, PCR and cloning

Total RNA was isolated using TriFast (Peqlab) according to the manufacturer's instruction. To produce cDNAs 1 μg of total RNA was reverse transcribed using oligo-dT18 primer and M-MLV reverse transcriptase (Promega). The cDNAs were used as template to amplify coding sequences or sequence parts of marker transcripts. The amplified fragments were analyzed on 1% agarose gels. The cDNAs coding for HMGA1a were produced by PCR using Phusion polymerase (NEB). Primers and PCR conditions can be delivered upon request to RH. All cDNAs were subcloned and verified by sequencing.

### Western blots

Cell nuclei were prepared as described [41]. 1.5 × 10^5 ^nuclei were loaded per lane and their proteins were separated on a 15% SDS-polyacrylamide gel. Unstained Protein Molecular Weight Marker (Fermentas) was used as a protein size standard. SDS-PAGE and transfer onto nitrocellulose was performed as described previously [41]. Loading and transfer efficiency was controlled by Ponceau S staining and appropriate Western blot controls. Blocking was carried out with 5% non-fat dry milk in TBST for 1 h. Nitrocellulose was washed three times in TBST for 10 min and incubated with the first antibody over night at 4°C. After three washing steps in TBST nitrocellulose membrane was incubated with the appropriate peroxidase conjugated secondary antibody in blocking solution. If necessary, nitrocellulose membrane was blocked for a second time in 5% non-fat dry milk/TBST prior to addition of the secondary antibody. Detection was performed by enhanced chemiluminescence as described [41]. Following antibodies were used for Western blot: anti-HMGA1 (1:2500, abcam), anti-GFP (1:1000, Roche), anti-MeCP2 (1:2000, Upstate), anti-HMGB1 (1:1000, Michael Bustin), anti-HMGN1 (1:1000, Micheal Bustin), anti-H1 (1:1000, Abcam), anti-H1 (1:500, Michael Bustin [43]) which was elicited against pure H1 subtractions and affinity purified against a mixture of all H1 sub-variants and anti Lamin A/C (1:5000, Santa Cruz).

## Authors' contributions

JB engineered and characterized the C2A1a cell line, carried out a substantial part of the analysis, interpretation of the data and helped to draft the manuscript. BV performed Western blots and helped to draft the manuscript. RH conceived of the study, designed the experiments, participated in data acquisition and drafted the manuscript. All authors read and approved the final manuscript.
